# Methyl 4-amino-3-meth­oxy­isoxazole-5-carboxyl­ate

**DOI:** 10.1107/S2414314623006235

**Published:** 2023-08-01

**Authors:** Mohd Abdul Fatah Abdul Manan, David B. Cordes, Alexandra M. Z. Slawin

**Affiliations:** a Faculty of Applied Sciences, Universiti Teknologi MARA, 40450 Shah Alam, Selangor, Malaysia; b EaStCHEM School of Chemistry, University of St Andrews, St Andrews, Fife KY16 9ST, United Kingdom; University of Aberdeen, United Kingdom

**Keywords:** crystal structure, isoxazole, hydrogen-bonded chain

## Abstract

The title compound, C_6_H_8_N_2_O_4_, a new derivative of isoxazole, has been synthesized and structurally characterized. The crystal structure shows the mol­ecule to be almost planar (r.m.s. deviation for the non-hydrogen atoms = 0.029 Å), this conformation being supported by an intra­molecular N—H⋯O hydrogen bond. In the extended structure, the mol­ecules are linked by N—H⋯O hydrogen bonds into chains propagating along [010].

## Abstract 2

The crystal structure of the title compound obtained from the reduction of its nitro precursor, methyl 3-meth­oxy-4-nitro­isoxazole-5-carboxyl­ate, is described.

## Graphical abstract

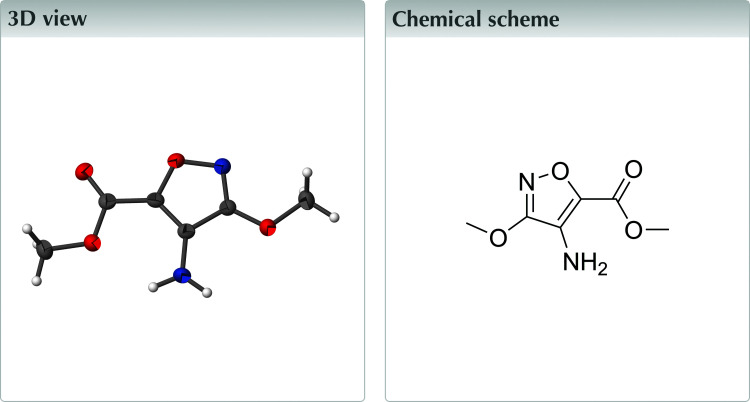

## Structure description

Isoxazoles, five-membered heterocyclic compounds containing adjacent nitro­gen and oxygen atoms, have many applications including in photochromic components (Pu *et al.*, 2011 ▸), liquid crystals (Kauhanka *et al.*, 2006 ▸), solar cells (Yoon *et al.*, 2022 ▸), high energy materials (Lal *et al.*, 2023 ▸), pesticides and insecticides (Wang *et al.*, 2022 ▸) and pharmaceuticals (Zhu *et al.*, 2018 ▸). In a continuation of our previous work on isoxazole derivatives (Abdul Manan *et al.*, 2023 ▸), we now present the synthesis and structure of the title compound.

**Figure 1:**
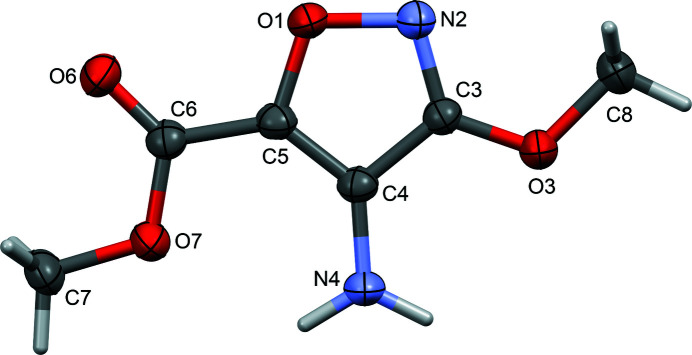
The mol­ecular structure of the title compound, showing displacement ellipsoids drawn at the 50% probability level.

The title compound, C_6_H_8_N_2_O_4_, crystallizes in space group *P*2_1_/*c* with one mol­ecule in the asymmetric unit (Fig. 1 ▸). All of the non-hydrogen atoms lie almost in the same plane, with an r.m.s. deviation of 0.029 Å and a maximum deviation of 0.060 (1) Å for C8. An intra­molecular N—H⋯O_e_ (e = ester) hydrogen bond (Table 1 ▸) helps to ensure the near co-planarity of the isoxazole and ester moieties. This whole-mol­ecule planarity, assisted by an intra­molecular hydrogen bond, is similar to what was observed in the related compounds ethyl 5-amino-3-methyl­isoxazole-4-carboxyl­ate (Sony *et al.*, 2005 ▸), ethyl 5-amino-3-(di­fluoro­meth­yl)isoxazole-4-carboxyl­ate (Schmitt *et al.*, 2015 ▸) and 5-amino-3-methyl­isoxazole-4-carbohydrazide (Regiec *et al.*, 2018 ▸). The relative orientation of the ester and amine groups, allowing the formation of the intra­molecular hydrogen bond to the ester oxygen atom rather than the carbonyl oxygen atom, is, however, different to what is seen in ethyl 5-amino-3-methyl­isoxazole-4-carboxyl­ate (Sony *et al.*, 2005 ▸), ethyl 5-amino-3-(di­fluoro­meth­yl)isoxazole-4-carboxyl­ate (Schmitt *et al.*, 2015 ▸), ethyl 5-amino-3-[fluoro(tri­fluorometh­oxy)meth­yl]isoxazole-4-carboxyl­ate (Schmitt *et al.*, 2017 ▸) and 1-(cyclo­hexyl­carbamo­yl)cyclo­hexyl 5-amino-3-methyl­isoxazole-4-carboxyl­ate (Bąchor *et al.*, 2019 ▸).

**Figure 2:**
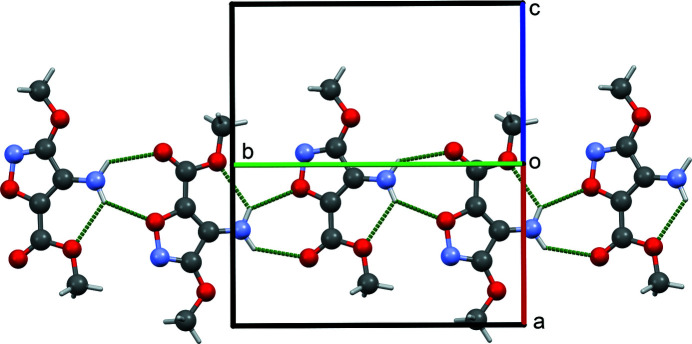
View down the [101] axis of the [010] chain formed by N—H⋯O hydrogen bonds, which are shown as dashed lines.

In the crystal of the title compound, adjacent mol­ecules are linked by N—H⋯O_c_ (c = carbon­yl) and N—H⋯O_i_ (i = isoxazole) hydrogen bonds, forming an 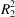 (7) loop, which generates chains of mol­ecules running along the crystallographic *b*-axis direction (Fig. 2 ▸). No additional directional inter­actions exist between chains. This combination of hydrogen bonds leading to chain formation is not seen in related isoxazole compounds as a result of the different relative position of the amine group on the isoxazole ring. While the combination of two inter- and one intra­molecular hydrogen bond has been seen previously in related isoxazoles (Sony *et al.*, 2005 ▸; Regiec *et al.*, 2018 ▸; Bąchor *et al.*, 2019 ▸), the pattern of hydrogen bonds is either different or has additional hydrogen bonds contributing to it, and the resulting supra­molecular motifs differ as well. One-dimensional chain motifs have been seen in two of the related isoxazoles (Schmitt *et al.*, 2015 ▸, 2017 ▸), although the pattern of hydrogen bonds that leads to the chains is different.

## Synthesis and crystallization

**Synthesis of the methyl 3-meth­oxy-4-nitro­isoxazole-5-carb­oxyl­ate precursor**

The starting material, methyl 3-meth­oxy­isoxazole-5-carboxyl­ate, was prepared according to the previously described literature procedure with minor modifications (Melikian *et al.*, 1992 ▸). K_2_CO_3_ (2.9 g, 21.0 mmol, 1.5 eq) and CH_3_I (1.3 ml, 21.0 mmol, 1.5 eq) were added to a solution of methyl 3-hy­droxy­isoxazole-5-carboxyl­ate (2.0 g, 13.9 mmol, 1.0 eq) in di­methyl­formamide (DMF) (10 ml) at 0°C. After 14 h stirring at room temperature, the mixture was poured into an ice-cold aqueous solution of HCl (0.5 *M*, 100 ml) and extracted into Et_2_O (5 × 80 ml). The combined organic layers were washed with a saturated aqueous solution of Na_2_CO_3_ (80 ml), dried over MgSO_4_, filtered and concentrated under reduced pressure to afford a light yellow crystalline solid, which was purified by silica gel column chromatography (petroleum ether/Et_2_O, 80:20), affording methyl 3-meth­oxy­isoxazole-5-carboxyl­ate (1.45 g, 66%) as a colourless crystalline solid.

Triflic anhydride (5.9 g, 21.0 mmol, 3.0 eq) was added to a solution of tetra­methyl­ammonium nitrate (2.9 g, 21.0 mmol, 3.0 eq) in DCM (3 ml) at room temperature. The suspension was stirred for 2 h, then a solution of methyl 3-meth­oxy­isoxazole-5-carboxyl­ate (1.1 g, 7.0 mmol, 1.0 eq) in di­chloro­methane (DCM) (10 ml) was added. After 48 h stirring under reflux, the mixture was cooled to room temperature and partitioned between water (30 ml) and DCM (40 ml). The organic layer was separated and washed with water (50 ml). The aqueous layer was extracted with DCM (3 × 50 ml). The combined organic layers were washed with brine (50 ml), dried over MgSO_4_, filtered and concentrated under reduced pressure. The resulting yellow residue was purified by silica gel column chromatography (petroleum ether/DCM, 50:50) to afford methyl 3-meth­oxy-4-nitro­isoxazole-5-carboxyl­ate (0.9 g, 70%) as yellowish oil: *R*_f_ = 0.41 (petroleum ether/Et_2_O, 80:20, UV/KMnO_4_); ^1^H (500 MHz, CDCl_3_), *δ*: (p.p.m): 4.14 (3*H*, *s*), 4.02 (3*H*, *s*); ^13^C (125 MHz, CDCl_3_), *δ*: (p.p.m): 164.0, 157.4, 155.0, 127.7, 58.9, 54.2; HRMS *m/z* (APCI^+^), found: [*M* + H]^+^ 203.0295, C_6_H_7_N_2_O_6_ requires [*M* + H]^+^ 203.0299.

**Synthesis of methyl 4-amino-3-meth­oxy­isoxazole-5-carboxyl­ate**

Iron powder (267 mg, 4.86 mmol, 5.0 eq) was added to a solution of methyl 3-meth­oxy-4-nitro­isoxazole-5-carboxyl­ate (196 mg, 0.97 mmol, 1.0 eq) in AcOH/H_2_O (AcOH = acetic acid) (3:1 *v*/*v* mixture, 12 ml). After stirring at 50°C for 2 h, the solution was cooled to room temperature and the solvent was removed under reduced pressure. The residue was partitioned between water (20 ml) and ethyl acetate (EtOAc) (20 ml). The mixture was basified with a saturated aqueous solution of Na_2_CO_3_ and further extracted with EtOAc (3 × 20 ml). The combined organic layers were washed with brine (20 ml), dried over MgSO_4_, filtered and concentrated under reduced pressure to afford a pale-yellow solid, which was purified by silica gel column chromatography (DCM, 100), affording methyl 4-amino-3-meth­oxy­isoxazole-5-carboxyl­ate (139 mg, 83%) as a colourless crystalline solid: *R*_f_ = 0.74 (DCM/EtOAc, 90:10, UV/ninhydrin); m.p. 111–112°C; ^1^H (500 MHz, CDCl_3_), *δ*: (p.p.m): 4.15 (*br s*, 2H), 4.05 (3*H*, *s*), 3.92 (3*H*, s); ^13^C (125 MHz, CDCl_3_), *δ*: (p.p.m): 164.5, 159.1, 138.4, 125.6, 57.5, 51.9; HRMS *m/z* (ESI^+^), found: [*M* + Na]^+^ 195.0373, C_6_H_8_N_2_O_4_Na requires [*M* + Na]^+^ 195.0382.

## Refinement

Crystal data, data collection and structure refinement details are summarized in Table 2 ▸.

## Supplementary Material

Crystal structure: contains datablock(s) I. DOI: 10.1107/S2414314623006235/hb4437sup1.cif

Structure factors: contains datablock(s) I. DOI: 10.1107/S2414314623006235/hb4437Isup2.hkl

Supporting information file. DOI: 10.1107/S2414314623006235/hb4437Isup3.mol

Supporting information file. DOI: 10.1107/S2414314623006235/hb4437Isup4.cml

CCDC reference: 2281787

Additional supporting information:  crystallographic information; 3D view; checkCIF report

## Figures and Tables

**Table 1:** Hydrogen-bond geometry (Å, °)

*D*—H⋯*A*	*D*—H	H⋯*A*	*D*⋯*A*	*D*—H⋯*A*
N4—H4*A*⋯O6^i^	0.89 (1)	2.30 (2)	2.9765 (16)	133 (1)
N4—H4*B*⋯O1^i^	0.91 (1)	2.31 (2)	3.0233 (15)	136 (1)
N4—H4*B*⋯O7	0.91 (1)	2.30 (2)	2.8734 (16)	121 (1)

Symmetry code: (i) 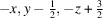 .

**Table 2:** Experimental details

Crystal data	
Chemical formula	C_6_H_8_N_2_O_4_
*M*_r_	172.14
Crystal system, space group	Monoclinic, *P*2_1_/*c*
Temperature (K)	173
*a*, *b*, *c* (Å)	7.0425 (18), 11.555 (3), 9.654 (2)
β (°)	106.629 (6)
*V* (Å^3^)	752.7 (3)
*Z*	4
Radiation type	Mo *K*α
μ (mm^−1^)	0.13
Crystal size (mm)	0.27 × 0.06 × 0.06

Data collection	
Diffractometer	Rigaku XtaLAB P200
Absorption correction	Multi-scan (*CrystalClear*; Rigaku, 2014 ▸)
*T*_min_, *T*_max_	0.695, 0.992
No. of measured, independent and observed [*F* ^2^ > 2.0σ(*F* ^2^)] reflections	9048, 1388, 1223
*R*_int_	0.051
(sin θ/λ)_max_ (Å^−1^)	0.604

Refinement	
*R*[*F* ^2^ > 2σ(*F* ^2^)], *wR*(*F* ^2^), *S*	0.030, 0.083, 1.09
No. of reflections	1388
No. of parameters	119
No. of restraints	2
H-atom treatment	H atoms treated by a mixture of independent and constrained refinement
Δρ_max_, Δρ_min_ (e Å^−3^)	0.17, −0.20

Computer programs: *CrystalClear* and *CrystalStructure* (Rigaku, 2014 ▸), *SIR2011* (Burla *et al.*, 2012 ▸), *SHELXL2018/3 (Sheldrick, 2015 ▸*), *Mercury* (Macrae *et al.*, 2020 ▸) and *publCIF* (Westrip, 2010 ▸).

## full crystallographic data

### Crystal data

**Table:**

C_6_H_8_N_2_O_4_	*F*(000) = 360.00
*M**_r_* = 172.14	*D*_x_ = 1.519 Mg m^−^^3^
Monoclinic, *P*2_1_/*c*	Mo *K*α radiation, λ = 0.71075 Å
*a* = 7.0425 (18) Å	Cell parameters from 2384 reflections
*b* = 11.555 (3) Å	θ = 2.8–25.4°
*c* = 9.654 (2) Å	µ = 0.13 mm^−^^1^
β = 106.629 (6)°	*T* = 173 K
*V* = 752.7 (3) Å^3^	Prism, colorless
*Z* = 4	0.27 × 0.06 × 0.06 mm

### Data collection

**Table:**

Rigaku XtaLAB P200 diffractometer	1388 independent reflections
Radiation source: Rotating Anode, Rigaku FR-X	1223 reflections with *F*^2^ > 2.0σ(*F*^2^)
Rigaku Osmic Confocal Optical System monochromator	*R*_int_ = 0.051
Detector resolution: 11.628 pixels mm^-1^	θ_max_ = 25.4°, θ_min_ = 2.8°
ω scans	*h* = −8→8
Absorption correction: multi-scan (CrystalClear; Rigaku, 2014)	*k* = −13→13
*T*_min_ = 0.695, *T*_max_ = 0.992	*l* = −11→11
9048 measured reflections	

### Refinement

**Table:**

Refinement on *F*^2^	Secondary atom site location: difference Fourier map
*R*[*F*^2^ > 2σ(*F*^2^)] = 0.030	Hydrogen site location: mixed
*wR*(*F*^2^) = 0.083	H atoms treated by a mixture of independent and constrained refinement
*S* = 1.09	*w* = 1/[σ^2^(*F*_o_^2^) + (0.0426*P*)^2^ + 0.1362*P*] where *P* = (*F*_o_^2^ + 2*F*_c_^2^)/3
1388 reflections	(Δ/σ)_max_ < 0.001
119 parameters	Δρ_max_ = 0.17 e Å^−^^3^
2 restraints	Δρ_min_ = −0.20 e Å^−^^3^
Primary atom site location: structure-invariant direct methods	

### Special details

**Table:**

Geometry. All esds (except the esd in the dihedral angle between two l.s. planes) are estimated using the full covariance matrix. The cell esds are taken into account individually in the estimation of esds in distances, angles and torsion angles; correlations between esds in cell parameters are only used when they are defined by crystal symmetry. An approximate (isotropic) treatment of cell esds is used for estimating esds involving l.s. planes.
Refinement. Refinement was performed using all reflections. The weighted R-factor (wR) and goodness of fit (S) are based on F^2^. R-factor (gt) are based on F. The threshold expression of F^2^ > 2.0 sigma(F^2^) is used only for calculating R-factor (gt).Carbon-bound H atoms were included in calculated positions (C—H = 0.98 Å) and refined as riding atoms with *U*_iso_(H) = 1.2*U*_eq_(C). The nitrogen-bound hydrogen atoms were located from difference Fourier maps and refined isotropically subject to a distance restraint.

### Fractional atomic coordinates and isotropic or equivalent isotropic displacement parameters (Å^2^)

**Table:**

	*x*	*y*	*z*	*U*_iso_*/*U*_eq_	
O1	−0.02039 (14)	0.26697 (7)	0.62559 (10)	0.0332 (3)	
O3	0.32430 (13)	0.09325 (8)	0.53449 (10)	0.0333 (3)	
O6	−0.30018 (14)	0.23906 (8)	0.77949 (11)	0.0376 (3)	
O7	−0.19751 (14)	0.05355 (8)	0.82206 (10)	0.0355 (3)	
N2	0.12840 (17)	0.25137 (9)	0.55608 (12)	0.0321 (3)	
N4	0.11557 (19)	−0.02825 (9)	0.71104 (14)	0.0380 (3)	
H4A	0.201 (2)	−0.0715 (13)	0.6805 (17)	0.044 (4)*	
H4B	0.039 (2)	−0.0582 (14)	0.7641 (17)	0.046 (4)*	
C3	0.18521 (18)	0.14406 (10)	0.58185 (13)	0.0275 (3)	
C4	0.08233 (18)	0.08329 (10)	0.66715 (13)	0.0271 (3)	
C5	−0.04431 (18)	0.16376 (10)	0.69055 (14)	0.0289 (3)	
C6	−0.19340 (18)	0.16034 (10)	0.76742 (14)	0.0297 (3)	
C7	−0.3398 (2)	0.03562 (12)	0.90147 (17)	0.0396 (4)	
H7A	−0.313327	0.089774	0.982892	0.048*	
H7B	−0.329201	−0.043968	0.937968	0.048*	
H7C	−0.473727	0.048846	0.837561	0.048*	
C8	0.4105 (2)	0.16335 (12)	0.44464 (16)	0.0375 (3)	
H8A	0.471816	0.232123	0.498567	0.045*	
H8B	0.306859	0.187186	0.357868	0.045*	
H8C	0.511435	0.118384	0.416398	0.045*	

### Atomic displacement parameters (Å^2^)

**Table:**

	*U*^11^	*U*^22^	*U*^33^	*U*^12^	*U*^13^	*U*^23^
O1	0.0415 (5)	0.0222 (4)	0.0413 (5)	0.0044 (4)	0.0205 (4)	0.0028 (4)
O3	0.0375 (5)	0.0275 (5)	0.0410 (6)	0.0027 (4)	0.0211 (4)	0.0030 (4)
O6	0.0383 (5)	0.0303 (5)	0.0495 (6)	0.0026 (4)	0.0209 (5)	−0.0031 (4)
O7	0.0399 (5)	0.0281 (5)	0.0451 (6)	−0.0013 (4)	0.0227 (4)	0.0007 (4)
N2	0.0385 (6)	0.0262 (6)	0.0360 (6)	0.0012 (4)	0.0178 (5)	0.0013 (4)
N4	0.0473 (7)	0.0223 (6)	0.0534 (8)	0.0053 (5)	0.0291 (6)	0.0077 (5)
C3	0.0299 (6)	0.0245 (6)	0.0291 (7)	−0.0004 (5)	0.0102 (5)	−0.0018 (5)
C4	0.0294 (7)	0.0231 (6)	0.0283 (6)	−0.0013 (5)	0.0077 (5)	−0.0013 (5)
C5	0.0340 (7)	0.0224 (6)	0.0309 (7)	−0.0018 (5)	0.0104 (6)	−0.0006 (5)
C6	0.0316 (7)	0.0260 (6)	0.0318 (7)	−0.0026 (5)	0.0098 (5)	−0.0042 (5)
C7	0.0421 (8)	0.0377 (8)	0.0469 (9)	−0.0057 (6)	0.0252 (7)	0.0002 (6)
C8	0.0398 (8)	0.0368 (7)	0.0427 (8)	0.0006 (6)	0.0227 (6)	0.0057 (6)

### Geometric parameters (Å, º)

**Table:**

O1—C5	1.3801 (15)	N4—H4B	0.910 (14)
O1—N2	1.4083 (15)	C3—C4	1.4275 (18)
O3—C3	1.3302 (15)	C4—C5	1.3514 (17)
O3—C8	1.4417 (16)	C5—C6	1.4493 (19)
O6—C6	1.2070 (15)	C7—H7A	0.9800
O7—C6	1.3456 (16)	C7—H7B	0.9800
O7—C7	1.4403 (17)	C7—H7C	0.9800
N2—C3	1.3048 (16)	C8—H8A	0.9800
N4—C4	1.3560 (17)	C8—H8B	0.9800
N4—H4A	0.893 (13)	C8—H8C	0.9800
			
C5—O1—N2	108.07 (9)	O6—C6—O7	124.71 (12)
C3—O3—C8	115.92 (10)	O6—C6—C5	126.27 (12)
C6—O7—C7	115.91 (10)	O7—C6—C5	109.01 (10)
C3—N2—O1	105.08 (10)	O7—C7—H7A	109.5
C4—N4—H4A	120.1 (11)	O7—C7—H7B	109.5
C4—N4—H4B	117.4 (11)	H7A—C7—H7B	109.5
H4A—N4—H4B	122.2 (15)	O7—C7—H7C	109.5
N2—C3—O3	124.73 (11)	H7A—C7—H7C	109.5
N2—C3—C4	113.51 (11)	H7B—C7—H7C	109.5
O3—C3—C4	121.76 (11)	O3—C8—H8A	109.5
C5—C4—N4	131.68 (12)	O3—C8—H8B	109.5
C5—C4—C3	103.07 (11)	H8A—C8—H8B	109.5
N4—C4—C3	125.24 (11)	O3—C8—H8C	109.5
C4—C5—O1	110.27 (11)	H8A—C8—H8C	109.5
C4—C5—C6	132.56 (12)	H8B—C8—H8C	109.5
O1—C5—C6	117.18 (11)		
			
C5—O1—N2—C3	−0.22 (13)	N4—C4—C5—C6	−1.7 (2)
O1—N2—C3—O3	179.64 (11)	C3—C4—C5—C6	179.23 (13)
O1—N2—C3—C4	−0.08 (14)	N2—O1—C5—C4	0.46 (14)
C8—O3—C3—N2	−2.12 (18)	N2—O1—C5—C6	−179.30 (10)
C8—O3—C3—C4	177.58 (11)	C7—O7—C6—O6	−0.80 (19)
N2—C3—C4—C5	0.35 (14)	C7—O7—C6—C5	−179.66 (10)
O3—C3—C4—C5	−179.38 (11)	C4—C5—C6—O6	−179.16 (14)
N2—C3—C4—N4	−178.81 (12)	O1—C5—C6—O6	0.53 (19)
O3—C3—C4—N4	1.45 (19)	C4—C5—C6—O7	−0.32 (19)
N4—C4—C5—O1	178.61 (13)	O1—C5—C6—O7	179.37 (10)
C3—C4—C5—O1	−0.48 (13)		

### Hydrogen-bond geometry (Å, º)

**Table:**

*D*—H···*A*	*D*—H	H···*A*	*D*···*A*	*D*—H···*A*
N4—H4*A*···O6^i^	0.89 (1)	2.30 (2)	2.9765 (16)	133 (1)
N4—H4*B*···O1^i^	0.91 (1)	2.31 (2)	3.0233 (15)	136 (1)
N4—H4*B*···O7	0.91 (1)	2.30 (2)	2.8734 (16)	121 (1)

Symmetry code: (i) −*x*, *y*−1/2, −*z*+3/2.
